# Influence of surface carbon on the performance of cesiated p-GaN photocathodes with high quantum efficiency

**DOI:** 10.1038/s41598-023-30329-0

**Published:** 2023-02-23

**Authors:** Jana Schaber, Rong Xiang, Jochen Teichert, André Arnold, Petr Murcek, Paul Zwartek, Anton Ryzhov, Shuai Ma, Stefan Gatzmaga, Peter Michel

**Affiliations:** 1 Institute of Radiation Physics (ELBE), Helmholtz Zentrum Dresden Rossendorf, Dresden, Germany; 2 Department of Physical Chemistry, Technische Universität Dresden, Dresden, Germany

**Keywords:** Physics, Condensed-matter physics, Surfaces, interfaces and thin films, Materials science, Condensed-matter physics, Semiconductors

## Abstract

This study shows residual surface carbon’s influence on photocathodes’ quantum efficiency based on p-GaN grown on sapphire by metal organic chemical vapor deposition. An X-ray photoelectron spectrometer (XPS) built in an ultrahigh vacuum system allowed the in-situ monitoring of the photocathode surface beginning immediately after their cleaning and throughout the activation and degradation processes. An atomically clean surface is necessary to achieve a negative electron affinity, which is the main prerequisite for high quantum efficiency. The p-GaN samples were cleaned with ethanol and underwent a sub-sequential thermal vacuum cleaning. Although carbon and oxygen contaminations are expected to be undesired impurities from the metal organic chemical vapor deposition, which remained on the surface, p-GaN could still form a negative electron affinity surface when exclusively activated with cesium. After the activation with cesium, a shift to a higher binding energy of the photoemission peaks was observed, and a new species, a so-called cesium carbide, was formed, growing over time. The XPS data elucidated the critical role of these cesium carbide species in photocathode degradation. The X-ray damage to the p-GaN:Cs photocathodes, especially the influence on the cesium, was additionally discussed.

## Introduction

Gallium nitride (GaN) has garnered increasing interest, especially since the Nobel Prize was awarded for developing GaN light-emitting diodes (LEDs) in 2014^1,2^. New opportunities for the use of III–V semiconductors and their alloys in photonic devices, data-storage applications, UV detectors, and high-power electronics arose, with the possibility of manipulating the semiconductor band gap by adding dopant atoms, such as indium (In), magnesium (Mg), and aluminum (Al) into the crystal lattice^3^. A novel application emerged from GaN’s ability to provide high-current electron beams for accelerator-based light sources, such as free-electron laser (FEL) and terahertz (THz) radiation sources. FEL and THz radiation sources are powerful tools for research in biomedicine, security imaging, and condensed matter physics^4,5^.

P-type GaN with Mg doping can form a negative electron affinity (NEA) surface when activated with an alkali metal, such as cesium (Cs). When used as a photocathode, the cesiated p-GaN can reach high quantum efficiency (QE) values of up to 70%^6–8^. The requirements for photocathodes for particle accelerator applications represent an extraordinary challenge with high expectations, for instance, a long lifetime, low thermal emittance, a fast response time, and high QE^9–11^. Recently, p-GaN has become increasingly popular as a potential electron source because it can be more robust than other semiconductor photocathodes. Further advantages of p-GaN are its simple activation and its possible rejuvenation because the required NEA surface can be achieved by activating commercially available p-doped GaN solely with Cs^12^. Indeed, p-GaN does not require molecular oxygen (O_2_) for its activation, making the activation process less complex than that of gallium arsenide (GaAs) photocathodes^13^. The formation of an NEA surface in the p-GaN band structure is necessary to help photoelectrons enter the vacuum more easily and thus achieve a high QE and a long lifetime. A clean surface could be an important prerequisite for the formation of the NEA surface. Usually strong acids, such as sulfuric acid (H_2_SO_4_) and hydrogen peroxide (H_2_O_2_), or hydrochloric acid (HCl) are used in the cleaning process^14–16^ for III–V semiconductors. However, this work shows that a simple cleaning with pure 99% ethanol (EtOH), followed by a thermal vacuum cleaning, represents an alternative cleaning method for p-GaN, achieving high QE and manageable in all laboratories.

Monitoring the surface chemistry of a photocathode is thus an important task, but also a challenging one considering the rigid operation conditions for the photocathodes. Ideally, the photocathode’s surface conditions should be monitored, beginning with its activation and throughout its aging and degradation, all of which are important processes for determining photoemission performance.

In this study, X-ray photoelectron spectroscopy (XPS) was used to monitor the composition of p-GaN surfaces. We demonstrated that a successful NEA surface, and thus a high QE, can be achieved if the commercially available p-GaN is cleaned solely with EtOH before activation. A high performance of 3–9% QE can be achieved despite the presence of some carbon (C) and oxygen (O) impurities remaining on the p-GaN surface (these impurities are expected to be from the metal organic chemical vapor deposition (MOCVD) process^17–19^). Based on the same method, we provided further insight into the activation, degradation, and reactivation processes of the p-GaN samples. Finally, our results allowed us to discuss the X-ray damage to the cesiated GaN surface.

## Results and discussion

Commercially available 5 µm thick p-GaN layers grown on sapphire (Al_2_O_3_) with MOCVD were used.

All samples were Mg doped and had the same carrier concentration in the range of 6 × 10^16^—1 × 10^17^ cm^-3^, provided by the supplier^20^.

### Surface cleaning

**Figure 1:**
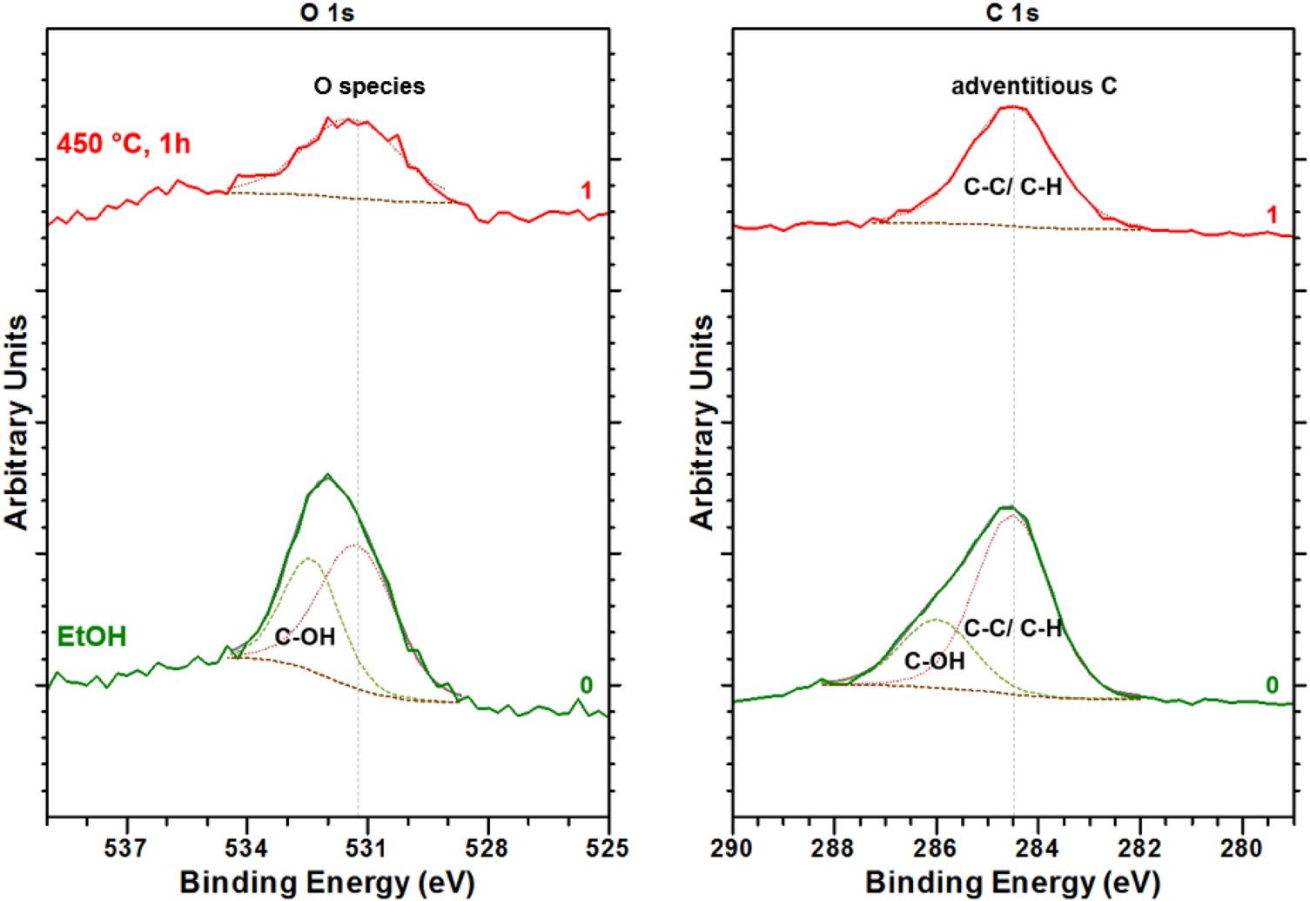
O 1 s and C 1 s photoelectron spectra for the p-GaN surface cleaned with EtOH (line 0) and after thermal cleaning at 450 °C (line 1). Dashed lines represent the peak fitting of the photoemission peaks.

Detailed XPS spectra of the O 1 s and C 1 s energy regions of the p-GaN surface rinsed in 99% EtOH (line 0) and after thermal cleaning at 450 °C (line 1) are shown in Fig. 1. The spectra were normalized to their backgrounds to have comparable intensities of the related photoemission peaks. All XPS measurements were conducted at room temperature.

The O 1 s photoemission peak is split into two components for the EtOH-cleaned p-GaN surface. The sub-peak at a binding energy (BE) of 531.4 eV was assigned to adsorbed molecular O_2_, while the O component at a BE at 532.2 eV derived to a weakly adsorbed hydroxyl (C–OH) compound^21,22^. The C 1 s photoemission peak showed the main C component at a BE of 284.5 eV derived from adventitious C^22,23^. In addition to this main C peak, a broad shoulder at 285.9 eV was observed originating from the EtOH’s C–OH components. These contaminations could have been reduced from their peak intensity when the p-GaN surface was thermally cleaned at 450 °C for 1 h, which is shown as line 1 in Fig. 1. The C–OH compound completely disappeared in both spectra, in the O 1 s and C 1 s, respectively. Thus, only a small but broad O peak with less intensity was observed after the thermal cleaning. It is assumed that this peak originated as an unwanted O impurity from an O species in the first sub-layers of the p-GaN lattice. The BE at 531.4 eV did not change for this O species, but its peak width was broader than before.

The C was not also entirely removed, but the peak intensity was reduced after the thermal cleaning. Thus, an atomically clean surface was not achieved.

Machuca et al*.* reported on the efficiency of a thermal vacuum cleaning at 700 °C leading in the successfully reduction of O and C contaminants on GaN surfaces^15^. Therefore, a series of additional experiments on p-GaN samples were carried out by a thermal vacuum cleaning at different temperatures between 320 and 650 °C. After the thermal cleaning, the presence of C and O was always observed on the p-GaN surface. Even at 650 °C it was not possible to remove C and O completely as a residual amount of C and O resulted in small, broad peaks as shown in Fig. S1 in the supplementary information.

Since the C and O remained on the p-GaN surface and were not removed completely by the thermal vacuum cleaning the p-GaN was additionally irradiated by Ar^+^ ions with 1.5 kV energy for 10 min. After the Ar^+^ irradiation, no C peak and only a small O peak was detectable on the p-GaN surface. Therefore, we assumed that the C was located at the surface and the O species might was located in the near sub-surface of p-GaN because the Ar^+^ were able to remove those contaminants.

However, temperatures above 500 °C and Ar^+^ irradiation were not considered for the following experiments because these treatments resulted in significant surface damage of p-GaN^12,24,25^.

Furthermore, external contamination sources inside the UHV chambers or during the sample transportation were excluded because the total pressure was always below 10^–11^ mbar, meaning that the partial pressures of the C and O species were below this level [measured by a residual gas analyzer (RGA SRS200)].

### Cs activation

**Figure 2:**
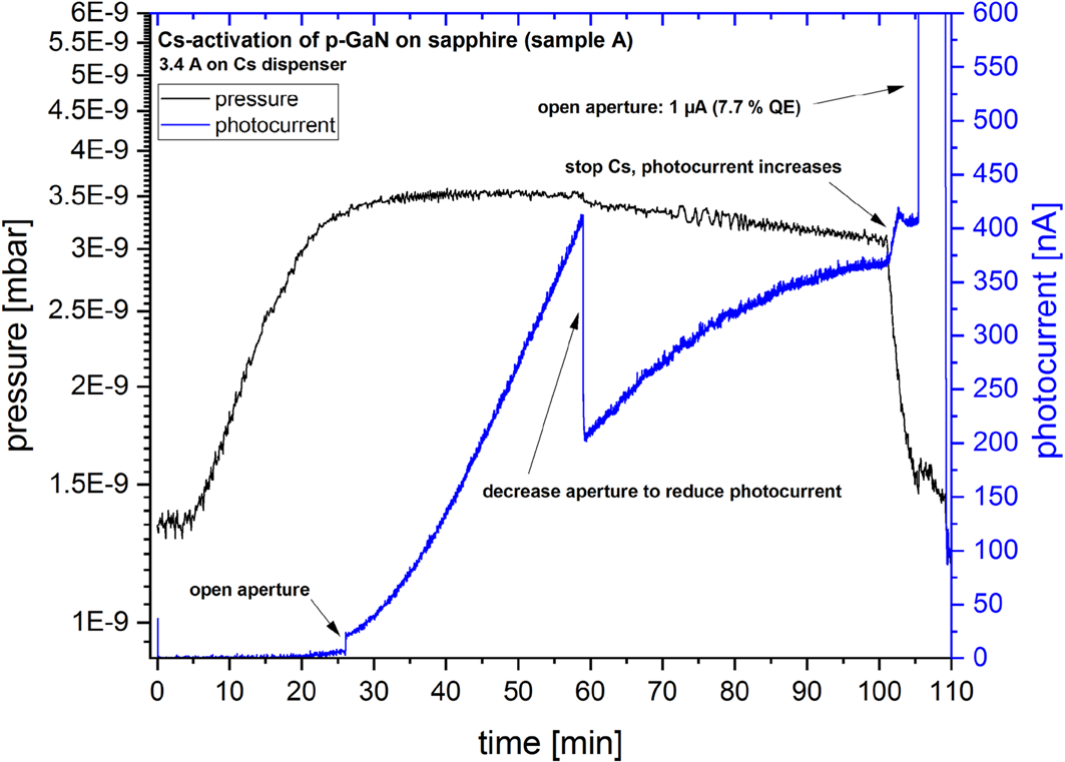
In-situ photocurrent (blue curve) and vacuum value (black curve, in logarithmic scale) during the Cs activation of the p-GaN surface (sample A).

A successful activation was possible by applying Cs to the p-GaN surface, although some C remained on the p-GaN surface after the thermal cleaning. The in-situ photocurrent, depicted in Fig. 2 as the blue curve, was measured during the Cs deposition. The vacuum in the ultrahigh vacuum (UHV) preparation chamber (the black curve) was maintained between 3.0 and 3.5 × 10^–9^ mbar due to a constant Cs flux. The photocurrent from the p-GaN:Cs photocathode was reduced by closing an aperture in front of the ultraviolet (UV) LED to keep the vacuum in the 10^–9^ mbar range. After approximately 100 min, when the photocurrent reached its maximum and began to saturate into a plateau, the Cs deposition was completed. The aperture was then fully opened to deliver the full photocurrent (1000 nA), corresponding to a QE of 7.7%, calculated with Eq. (1).

**Figure 3:**
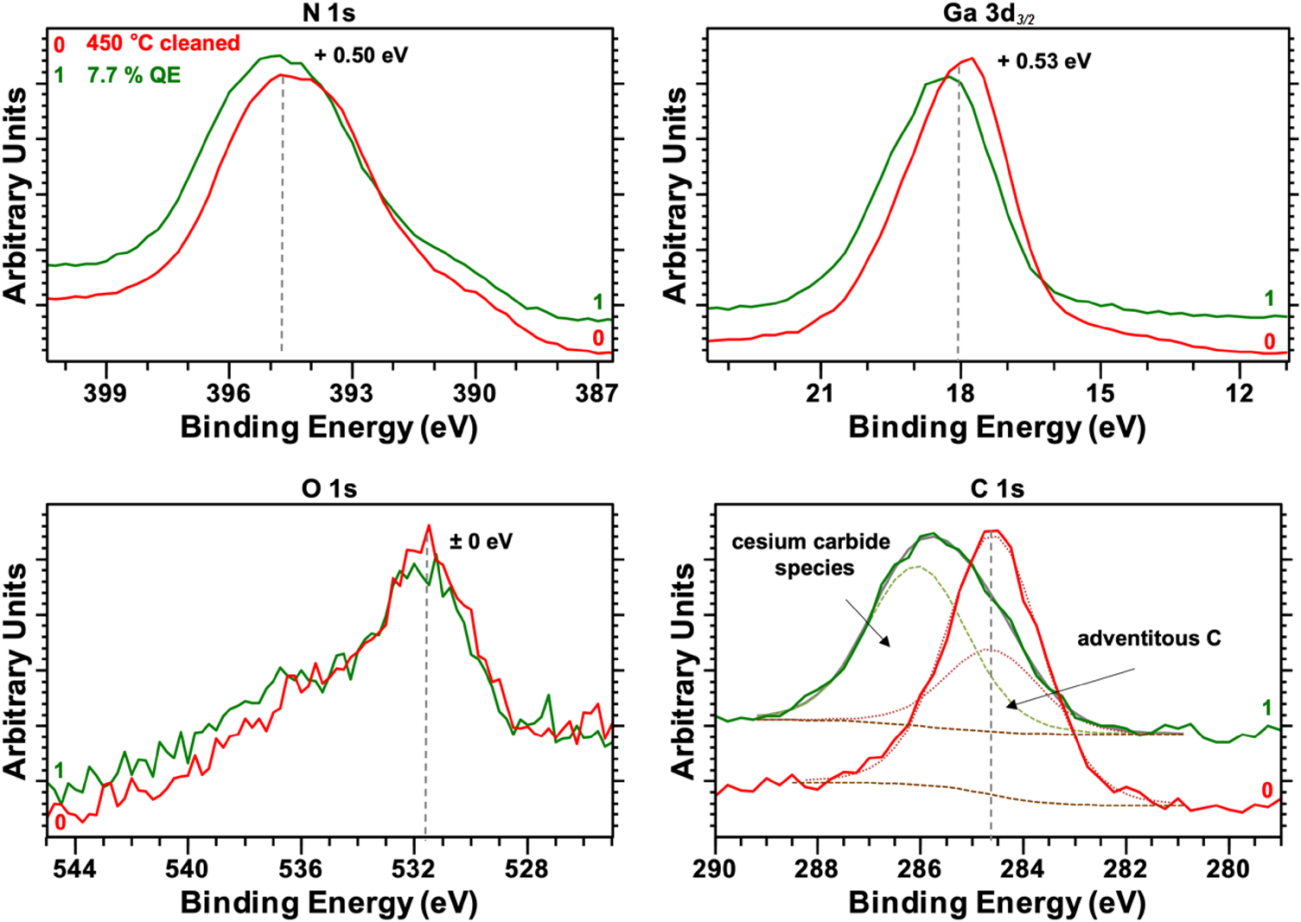
Ga 3d_*3/2*_, N 1 s, O 1 s, and C 1 s photoelectron spectra for the p-GaN surface after thermal cleaning at 450 °C (line 0) and after Cs activation with 7.7% QE (line 1). Dashed lines represent the peak fitting in the C 1 s spectra.

XPS analysis was used to study the effect of the Cs deposition on the chemical composition of the p-GaN surface. The N 1 s, O 1 s, C 1 s, and Ga 3d_*3/2*_ photoemission peaks are depicted in Fig. 3 to compare the p-GaN surface before (line 0) and after (line 1) the Cs activation was applied.

For the N 1 s and Ga 3d_*3/2*_ peaks, a shift of approximately 0.50 eV to a higher BE was observed. The electron density for these photoemission peaks changed due to the high electron input from the Cs on the p-GaN’s surface. This shift indicated the successful adsorption of the Cs on the p-GaN. No shift in the BE for O 1 s was observed, meaning that the detected O was not influenced by the Cs deposition and thus might be located in deeper surface sub-layers. The C 1 s photoemission peak was more strongly influenced by the deposition of Cs. As shown in the previous section, adventitious C was still present after the thermal cleaning and could not be removed entirely from the p-GaN surface. It is well known that undesired O and C impurities, in particular, are incorporated in the near-surface layers of the p-GaN crystal lattice during MOCVD growth^17–19^.

However, a new C component appeared at a BE of approximately 286 eV when the Cs was deposited. A small sub-peak, originating from adventitious C, was still detectable but decreased in intensity. Thus, the component at a higher BE (approximately 286 eV) must have been C surrounded by a higher electron density. We assumed that this new component showed the formation of a cesium carbide (Cs_x_C_y_) species. The freshly deposited Cs appeared more attracted to the residual C than to the p-GaN surface. A similar effect of the C photoemission peak shifting toward a higher BE depending on the Cs amount was reported for the adsorption of Cs on graphite^26^. To the best of our knowledge, there are few studies on alkali metal carbides.

**Figure 4:**
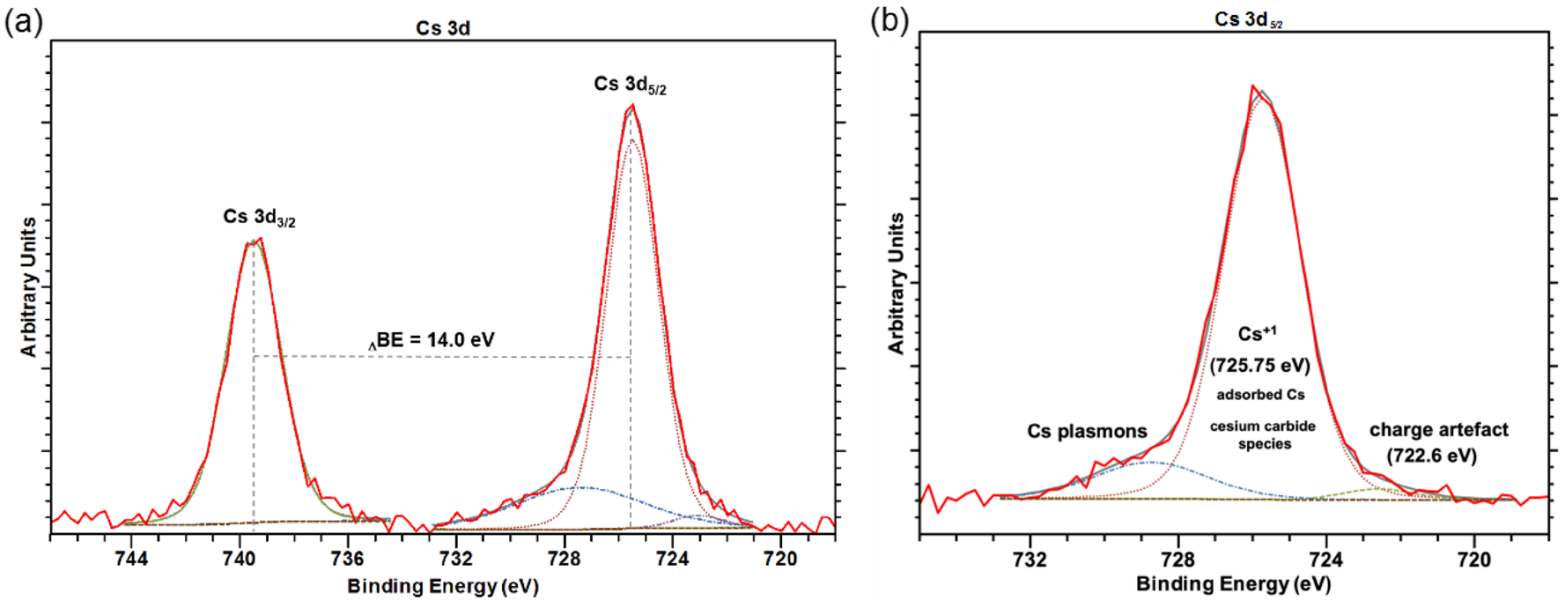
(**a**) Cs 3d photoemission spectrum of the activated p-GaN surface, showing the spin–orbit splitting at 14 eV and (**b**) Cs 3d_*5*/2_ photoemission spectrum of the adsorbed Cs on the p-GaN surface, with the peak fitting showing three components. The dashed lines represent the peak fitting.

Detailed XPS spectra were also taken in the Cs 3d core level region. They exhibited a typical paired structure due to spin–orbit splitting (739.75 eV for 3d_*3/2*_ and 725.75 eV for 3d_*5/2*_), as shown in the survey spectrum in Fig. 4a. The resulting spin–orbit shifted exactly 14 eV between corresponding peaks. Only the Cs 3d_*5/2*_ photoemission peak was considered for further evaluation. The 3d_*5/2*_ photoemission peak shifted toward a lower BE with respect to the free metallic Cs (Cs 3d_*5/2*_ = 726.4 eV), as shown in Fig. 4b^22^. The main Cs peak of 725.75 eV represented the adsorbed Cs on the p-GaN surface. The surface Cs was expected to be positively charged (Cs^+1^) in relation to the bulk Cs (Cs^0^). This shift toward a lower BE for Cs adsorbates has been previously observed and discussed^27,28^. Furthermore, a few publications have noted oxidization states of Cs oxides and the Cs peak shifting toward lower BE when the oxidization state changed to + 1 (Cs^+1^)^29–31^. The O 1 s photoemission peak usually appears at a BE of about 530 eV, within the typical BE range of an alkali metal oxide^32^. In contrast, in our study, the O 1 s peak was not influenced by the Cs. Therefore, we assumed that the Cs was attached to the p-GaN surface in a Cs^+1^ chemical state or as a Cs_x_C_y_, which must also be in a Cs^+1^ state. A broad peak at 729 eV was identified from Cs plasmons^22^. A further sub-peak at 722.6 eV could have originated from a charging artefact^33^, but its appearance is rarely reported.

### Degradation

Photocathodes undergo natural degradation caused by residual gases in a vacuum environment^34^. Even in an ideal UHV environment, photocathodes lose QE. Thus, the surface chemical state under UHV conditions at different times during storage was studied to investigate the correlation between the mechanism of cathode degradation and the surface chemical state change. A detailed XPS analysis allowed us to identify and quantify the relative elemental composition of the surface and distinguish among the main elements’ chemical bonding states.

**Figure 5:**
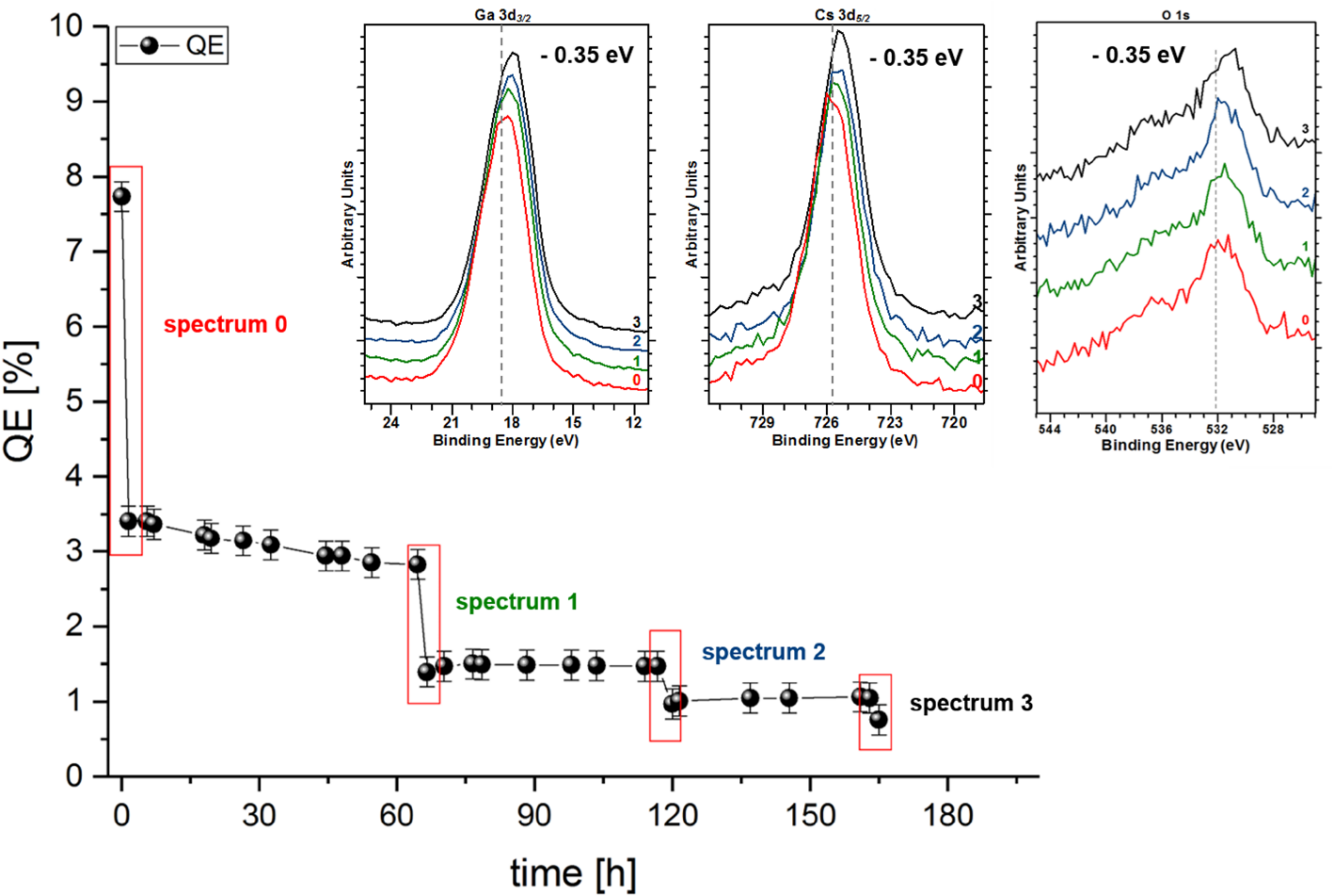
The QE decay of the p-GaN:Cs photocathode (sample A) and the Ga 3d_*3/2*_, Cs 3d_*5/2*_, and O 1 s photoemission spectra at different times during its decay.

Different photoemission peaks from the p-GaN:Cs photocathode were acquired at different stages of QE decay, as shown in Fig. 5. The freshly prepared photocathode initially had a 7.7% QE. A QE drop accompanied each XPS measurement due to X-ray damage, which is discussed in a later section. Between the XPS measurements, the QE decayed exponentially, as is typical of this material.

Figure 5 indicates that a shift of about 0.35 eV toward a lower BE was measured for Ga 3d_*3/2*_, Cs 3d_*5/2*_, N 1 s, O 1 s and C 1 s, along with the QE decay. The N 1 s spectra are not shown here because the Al excitation (see “Methods”) is ineffective for N 1 s analysis due to the superposition of the photoemission N 1 s line and the Auger Ga (the L_2_M_23_M_23_ auger process) transition^22^.

In general, a shift in the BE means that the electronic structure of the observed element changes, and its oxidization state subsequently changes. Therefore, the BE is often regarded as a “chemical shift”^35^. Furthermore, the dopant concentration and thus the Fermi-level location in semiconductors affects the BE’s location^36^ and hard X-ray irradiation can introduce a band bending^37^.

**Figure 6:**
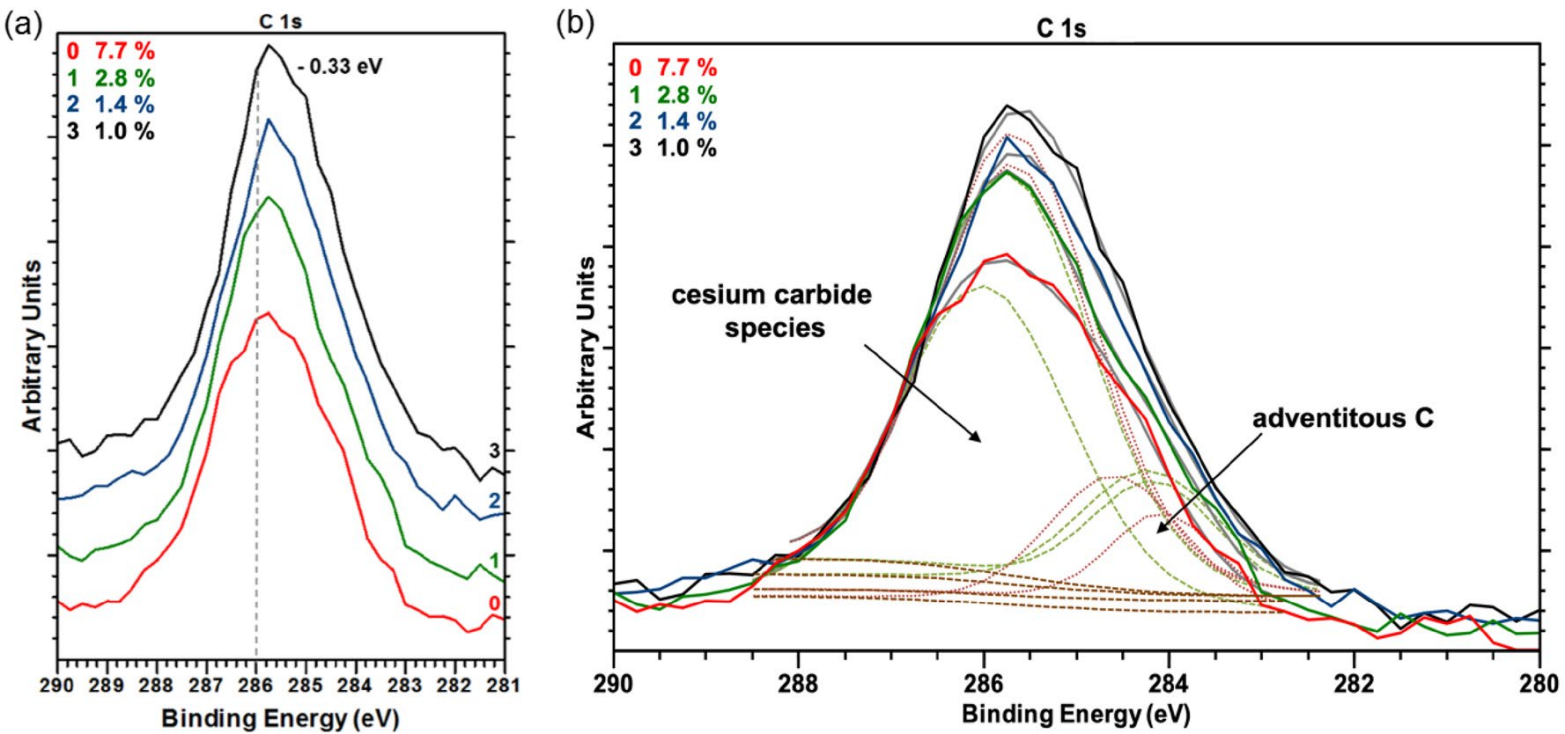
(**a**) Stacked C 1 s photoemission spectra showing the shift to lower BE along with the degradation of the p-GaN:Cs photocathode (sample A) and (**b**) the same C 1 s photoemission spectra (normalized to their background) showing the evolution of the cesium carbide (Cs_x_C_y_) species with peak fittings (dashed lines) along with the degradation.

The relative concentration of Cs did not change during the photocathode degradation; thus, we excluded a loss of Cs. No additional Cs compound was found, which implied that the Cs had not changed its chemical state. At the C 1 s photoemission peak, the intensity of the Cs_x_C_y_ species, at a BE of approximately 286 eV, increased significantly, and its peak width became narrower during QE decay, as shown in Fig. 6. We assumed that the adhesion between Cs and the p-GaN surface became weaker, which was reflected by the shift toward lower BE. This shift also indicated that the surface was still positively charged and that charging effects might occur^21^. Consequently, the GaN-Cs bonding became less pronounced, and the electron affinity increased until Cs_x_C_y_ dominated the p-GaN:Cs photocathode surface, disturbing the NEA surface, which disappeared. The possible reasons for changing an XPS’s peak intensity are typically (i) a change in the amount of atoms with the respective oxidative state associated with the surface or (ii) a change in the depth distribution of such atoms caused by diffusion processes^35^.

The XPS measurements indicate a growth of the amount of Cs_x_C_y_ species during the degradation process. Since the diffusion of C atoms from the bulk toward the surface in GaN has been reported^38^, we consider that such C diffusion could result in the formation of additional Cs_x_C_y_ compounds. To the best of our knowledge, the formation of Cs_x_C_y_ during the operation of Cs-activated photocathodes has not been previously reported. Accordingly, we can only suggest that the previously mentioned explanations must be considered cautiously. This phenomenon should be considered when working on p-GaN photocathodes, as it may be one of the critical factors determining the QE and these systems’ lifetimes.

**Figure 7:**
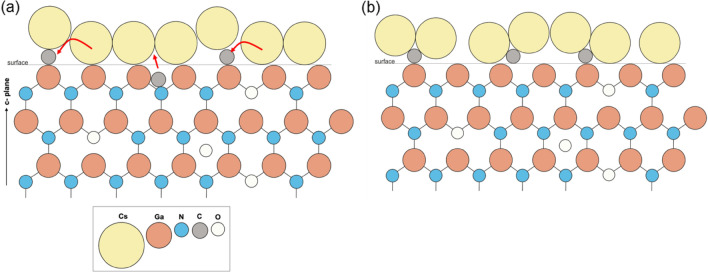
(**a**) The surface model of a freshly prepared p-GaN:Cs photocathode. The surface C attracts a part of the Cs and (**b**) the surface model of an aged p-GaN:Cs photocathode showing the formation of Cs_x_C_y_.

A surface model of a freshly prepared p-GaN:Cs photocathode is schematically illustrated in Fig. 7a. This model indicated that the formation and growth of Cs_x_C_y_ species caused the QE degradation. The p-GaN has a hexagonal crystal lattice that grows along the c-plane (0001), and the surface is mostly Ga terminated. In addition, O might have been present in the near-surface layers and C at the near surface, both as unwanted impurities from the MOCVD process^18,19^.

Trimethylgallium (Ga(CH_3_)_3_) is used as a precursor for Ga in the MOCVD process and high temperatures are necessary for its decomposition. Gow et al*.* showed that the decomposition of Ga(CH_3_)_3_ depended on the applied temperature and that a low C concentration could be detected in semiconductor sub-layers^17^. The residual C concentration reduction could be controlled during crystal growth, but C could not be removed entirely. The concentration of C associated with the surface was always higher than in the bulk^18,19^. Thus, we did not achieve an atomically clean surface, as some residual C from the growth process was always present. When Cs was applied, it was deposited on the p-GaN surface next to and on top of the C contaminants. With increasing storage time, the Cs atoms began moving toward the C atoms, and the adhesion between Cs and p-GaN became weaker, as illustrated in Fig. 7b. The C began to form a bond with Cs, that is, a Cs_x_C_y_ species. We assumed that in this process, some Cs_x_C_y_ islands were formed, which caused the QE of the photocathode to degrade.

### Reactivation

**Figure 8:**
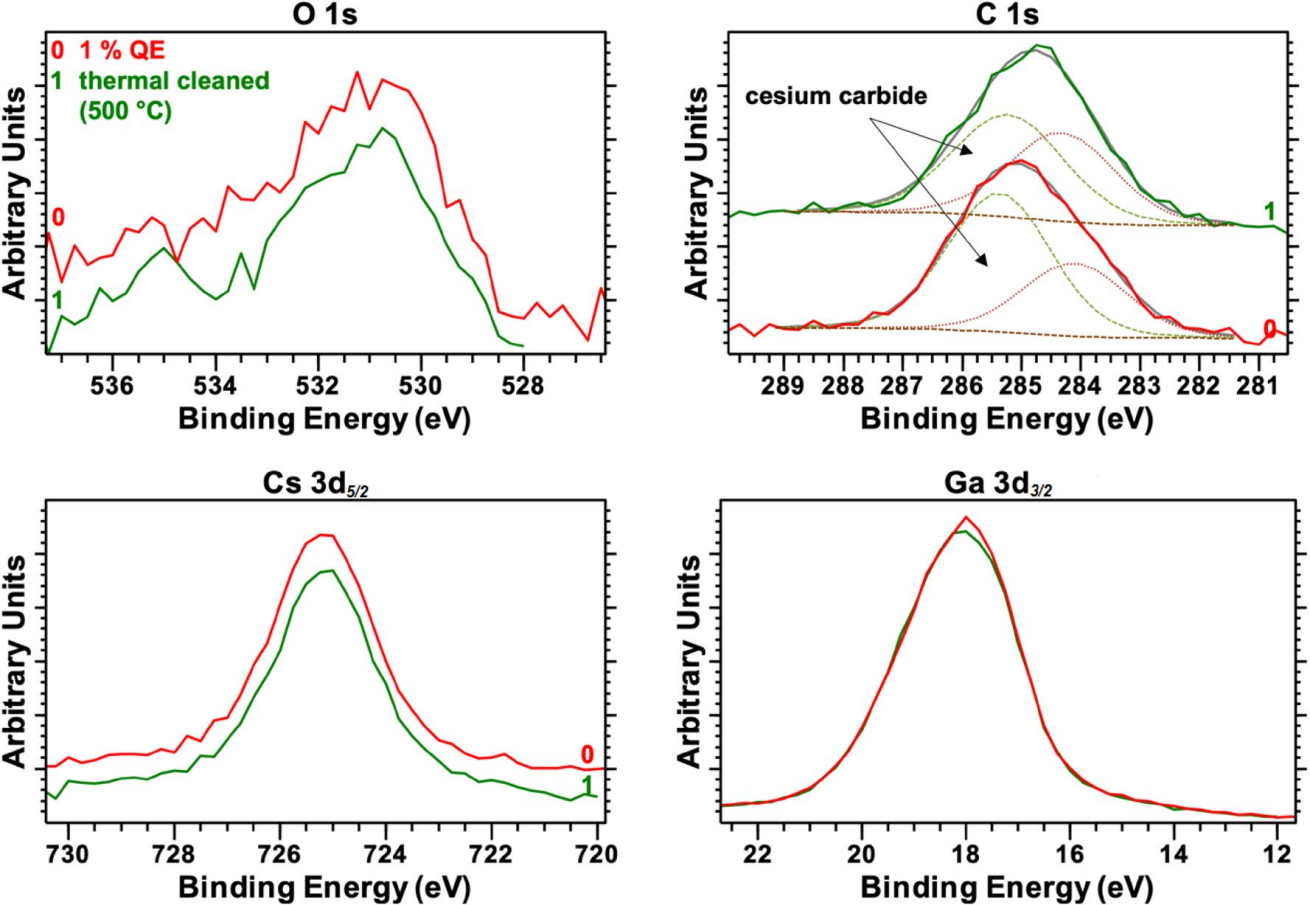
The Ga 3d_*3/2*_, Cs 3d_*5/2*_, O 1 s, and C 1 s photoelectron spectra of the p-GaN:Cs surface before (line 0) and after (line 1) renewed thermal cleaning at 500 °C. Dashed lines represent the peak fitting in the C 1 s spectra.

When the QE had a value below 1%, sample A was thermally cleaned before the subsequent Cs activation. After the new thermal cleaning, the photocurrent was partially restored even before the subsequent Cs activation was conducted. In a previous publication, we noted that this phenomenon occurred during reactivation, but it could only occur if Cs remained on the p-GaN’s surface^12^. As shown in Fig. 8, the XPS data confirmed that the Cs was not entirely removed from the p-GaN surface after the thermal cleaning.

A comparison of the photoemission peaks from sample A (with 1% remaining QE) before (line 0) and after (line 1) the thermal cleaning at 500 °C is shown in Fig. 8. For O 1 s and Ga 3p_*3/2*_, no difference before and after the thermal treatment was observed. The thermal cleaning also did not affect the Cs peak because it remained on the p-GaN surface and did not decrease in intensity or atomic concentration.

The most substantial effect of the thermal cleaning was again observed at the C 1 s peak, especially for the Cs_x_C_y_ species. The intensity of the Cs_x_C_y_ species decreased after the thermal cleaning, vice versa the intensity of adventitious C increased.

The sample was thermally cleaned at 500 °C for 120 min without successfully removing all the Cs and the Cs_x_C_y_ species. We assumed that during the thermal cleaning of the p-GaN:Cs photocathode, the Cs_x_C_y_ species decomposed into metallic Cs and adventitious C.

The p-GaN was reactivated with the deposition of freshly applied Cs, although some remaining Cs from the previous activation remained on the p-GaN’s surface. This time, the p-GaN:Cs photocathodes reached a QE of 9.2%, which was slightly higher than in the first activation. Thus, the old Cs that remained on the p-GaN surface did not negatively impact the reactivation process. It was in fact beneficial and led to a higher QE.

**Figure 9:**
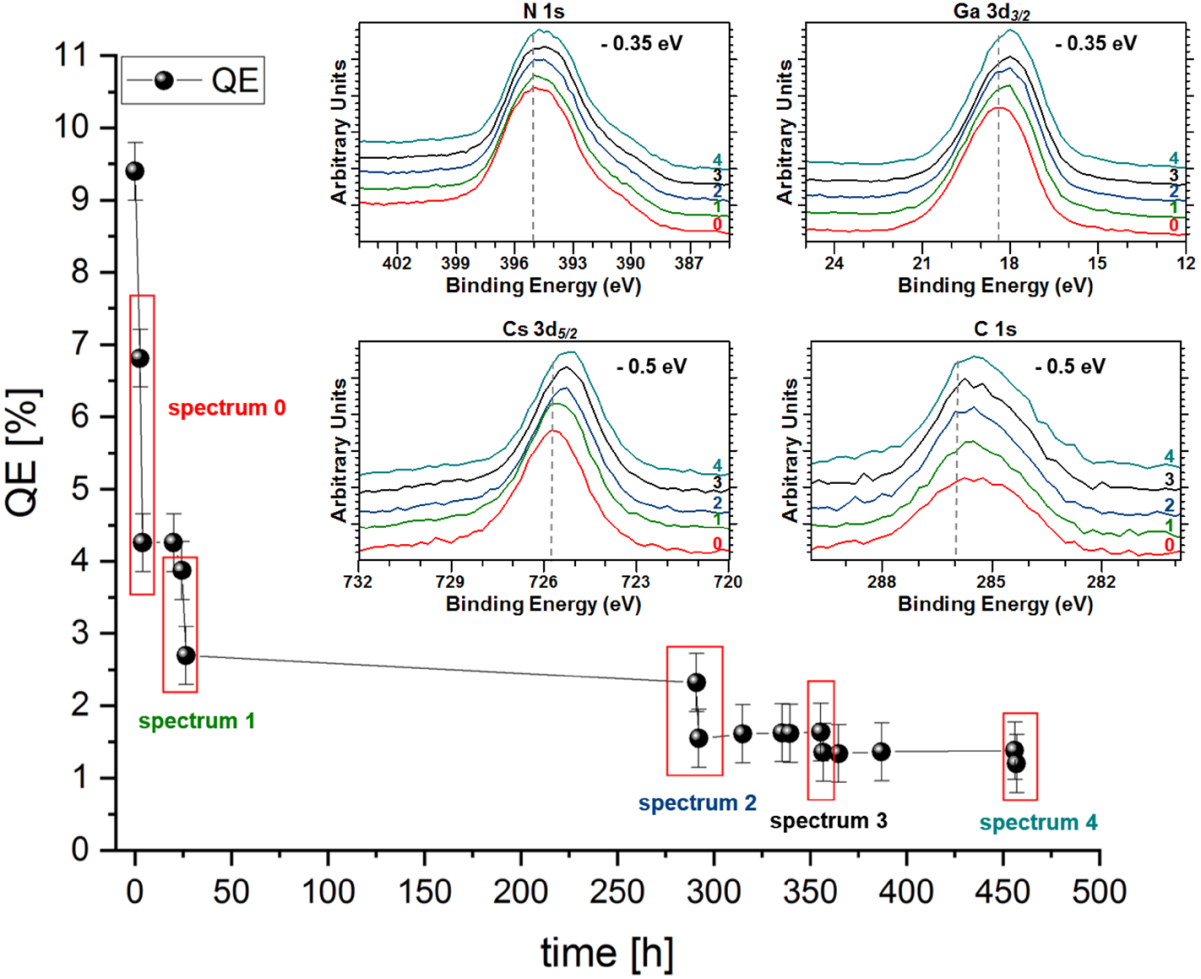
The QE decay curve of the reactivated p-GaN:Cs photocathode (sample A) with the corresponding N 1 s, Ga 3d_*3/2*_, Cs 3d_*5/2*_, and C 1 s photoemission spectra, taken at different points in the decay curve.

The degradation of the reactivated p-GaN:Cs photocathode, followed by XPS analysis, is shown in Fig. 9. The same effect in the degradation was observed, as explained in the previous section. The N 1 s and Ga 3d_*3/2*_ peaks shifted 0.35 eV, whereas the Cs 3d_*5/2*_ and C 1 s peaks shifted 0.5 eV toward a lower BE. These significant shifts indicated that the Cs and C amounts might correlate and that their behavior primarily caused the degradation.

To ensure the reproducibility of our results, two more p-GaN on sapphire samples (samples B and C) underwent similar treatments, and their XPS spectra are shown in Figs. S2–S7 in the supplementary information. Similar behaviors for these samples were also observed during activation, degradation, and reactivation.

### X-ray damage

The p-GaN:Cs photocathodes lost QE after every transport into the XPS analysis chamber. The QE decay curve for sample A is shown as an example in Fig. 5. The QE dropped significantly during the hours following activation, especially after the sample was analyzed by XPS. Typically, the QE decays exponentially for p-GaN:Cs photocathodes, as we indicated in another publication^12^. The QE stabilized when the sample was returned to the UHV preparation chamber. We found that the fast QE loss after the XPS analysis was not the result of transportation but was likeliest caused by the X-ray’s influence from the dual anode. In the following section, we provide more detail on this phenomenon.

The studies on the effects of X-rays during XPS analysis were conducted using a lengthy irradiation experiment. For this purpose, p-GaN (sample D) was treated the same way as samples A–C: it was cleaned with 99% pure EtOH, thermal cleaned for 1 h, and activated with Cs. The resulting p-GaN:Cs photocathode showed 7% QE after the Cs deposition. Sample D was then irradiated for 5.5 h with X-rays from the Mg source at a beam power of 100 W and 11 keV. The photocathode was neither transported nor moved. Thus, transportation did not influence these experiments.

**Figure 10:**
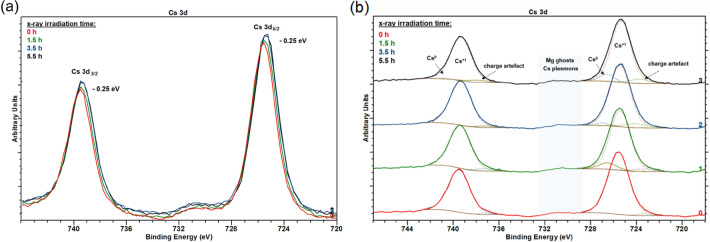
(**a**) Cs 3d photoelectron spectra for p-GaN:Cs surface (sample D) influenced by the X-ray’s irradiation time and (**b**) the same Cs 3d spectra of sample D, stacked and with peak fittings (dashed lines).

The Cs 3d_*5/2*_ and Cs 3d_*3/2*_ spin–orbit photoemission peaks, in relation to the X-ray exposure time, are shown in Fig. 10a. A shift of 0.25 eV toward a lower BE with rising irradiation time was observed. The shape of the Cs 3d peaks and the Cs concentration remained constant during this experiment.

Detailed spectra of the lengthy experiment with suitable fittings to the Cs 3d photoemission peaks are shown in Fig. 10b. The main peak at 725.5 eV was derived from adsorbed Cs with a positive charge (Cs^+1^) on the p-GaN surface, while the sub-peak at approximately 723 eV belonged to a charge artefact, as noted in the previous sections. When the irradiation time rose in lines 1–3, a Cs component of approximately 726 eV appeared in the photoemission spectra that was not observed for the freshly prepared photocathode (line 0). This photoemission peak at about 726 eV originated from free Cs (Cs^0^). The X-ray irradiation caused surface melting and unwanted heating, which resulted in a change in surface composition. Thus, the bulk Cs^0^ component was not observable at the beginning of the experiment when the photocathode was freshly prepared. The X-ray beam thermalized the sample and caused external aging of the photocathode. Furthermore, the Cs concentration did not change during an X-ray exposure of 5.5 h. We assumed that redistribution in the sample strongly influenced the adhesion between GaN and Cs. It is well known that X-ray irradiation significantly impacts semiconductors and their surfaces^35^. Consequently, the adsorbed Cs on p-GaN could be redistributed under X-ray heating, building metallic Cs. Nevertheless, we neither cooled nor measured the sample’s temperature. An X-ray beam power reduction or a sample’s cooling down could have prevented or at least decreased the X-ray damage.

Sample D had 7% QE before XPS analysis and showed only 1.2% QE after being treated by X-ray irradiation for 5.5 h. We assumed the X-ray damage also caused the QE losses observed in samples A–C. Furthermore, transportation effects, that might negatively influence QE decay in these experiments, were excluded. We recommend considering this X-ray damage risk when working with cesiated semiconductor photocathodes with X-ray-based analytical devices (XPS or X-ray diffraction [XRD]).

## Conclusion

We succeeded in cleaning and activating solely with Cs commercially available MOCVD grown p-GaN samples. A cleaning in 99% EtOH followed by a thermal cleaning at 450 °C in a vacuum represented a simple cleaning procedure, manageable in all laboratories. Each treatment step was followed by XPS analysis without leaving the UHV environment, revealing residual O and C on the p-GaN surface. Thus, a thermal treatment under vacuum did not entirely remove these organic contaminations, but the thermal cleaning reduced their peak intensities. The remaining O and C contaminations were assumed to be residuals derived from the MOCVD process.

We showed that activation exclusively with Cs was feasible and reliable. QE values of 3–9% were achieved when the p-GaN was activated. After activation, a shift toward a higher BE was observed in the XPS spectra of the related photoemission peaks. This shift indicated that the Cs was successfully adsorbed to the p-GaN surface. Before the Cs activation, adventitious C at a BE of approximately 284 eV was found, which was also present after the Cs activation but did not shift in its BE. We also showed that the presence of remaining C significantly influenced the photocathode’s quality. After the Cs deposition, a new C species at a higher BE (about 286 eV) in the C 1 s spectrum appeared. This new species showed a higher BE than adventitious C and was identified as a Cs_x_C_y_ species. With the ongoing degradation of the photocathode, the photoemission intensity of the Cs_x_C_y_ species increased. The formation and steady growth of the Cs_x_C_y_ species on the p-GaN surface were assumed to cause a shift of the related photoemission peaks to a lower BE. On the Cs 3d photoemission spectra, Cs showed a Cs^+1^ oxidization state, which had a lower BE than that of the metal Cs^0^. Thus, the Cs was positively charged when absorbed on the p-GaN surface and had a + 1 state when forming a Cs_x_C_y_.

We measured residual photocurrents after the samples were thermally cleaned before the next Cs activation. The XPS measurements revealed that the Cs was not entirely removed from the surface, and the remaining Cs seemed beneficial because higher QE values were achieved in the subsequent reactivations.

Under static conditions, the QE of photocathodes decays exponentially. We observed though an immense QE loss after the p-GaN:Cs photocathodes were studied by XPS. Furthermore, we determined that the origin of the QE loss derived primarily from the X-ray irradiation rather than by the sample’s transportation. Therefore, we investigated potential X-ray damage to the p-GaN:Cs photocathodes. These experiments showed that the adsorbed Cs and its adhesion to the p-GaN surface were strongly influenced by X-ray irradiation. The Cs photoemission peaks shifted toward a lower BE, while the relative Cs concentration did not. This shift indicated that X-ray irradiation accelerated the degradation of the surface conditions of the p-GaN photocathodes. Thus, we recommend using lower X-ray beam power or cooling the samples to prevent X-ray damage to cesiated photocathodes.

In this study, we achieved high QE values for p-GaN:Cs on sapphire photocathodes, although C and O appear to be incorporated as unwanted impurities in the near-surface layers of the p-GaN lattice due to MOCVD. The undesired C concentration was low enough to allow the formation of an NEA surface and thus obtain a p-GaN:Cs photocathode with high QE. The surface measurements using XPS indicated that the undesired formation of Cs_x_C_y_ on the surface caused degradation of the photocathodes. We assume that the photocathode performance, especially the QE, could be improved by using a higher purity p-GaN crystal with ideally no C and O impurities incorporated in the near-surface layers inside the p-GaN lattice.

## Methods

### Experimental setup

**Figure 11:**
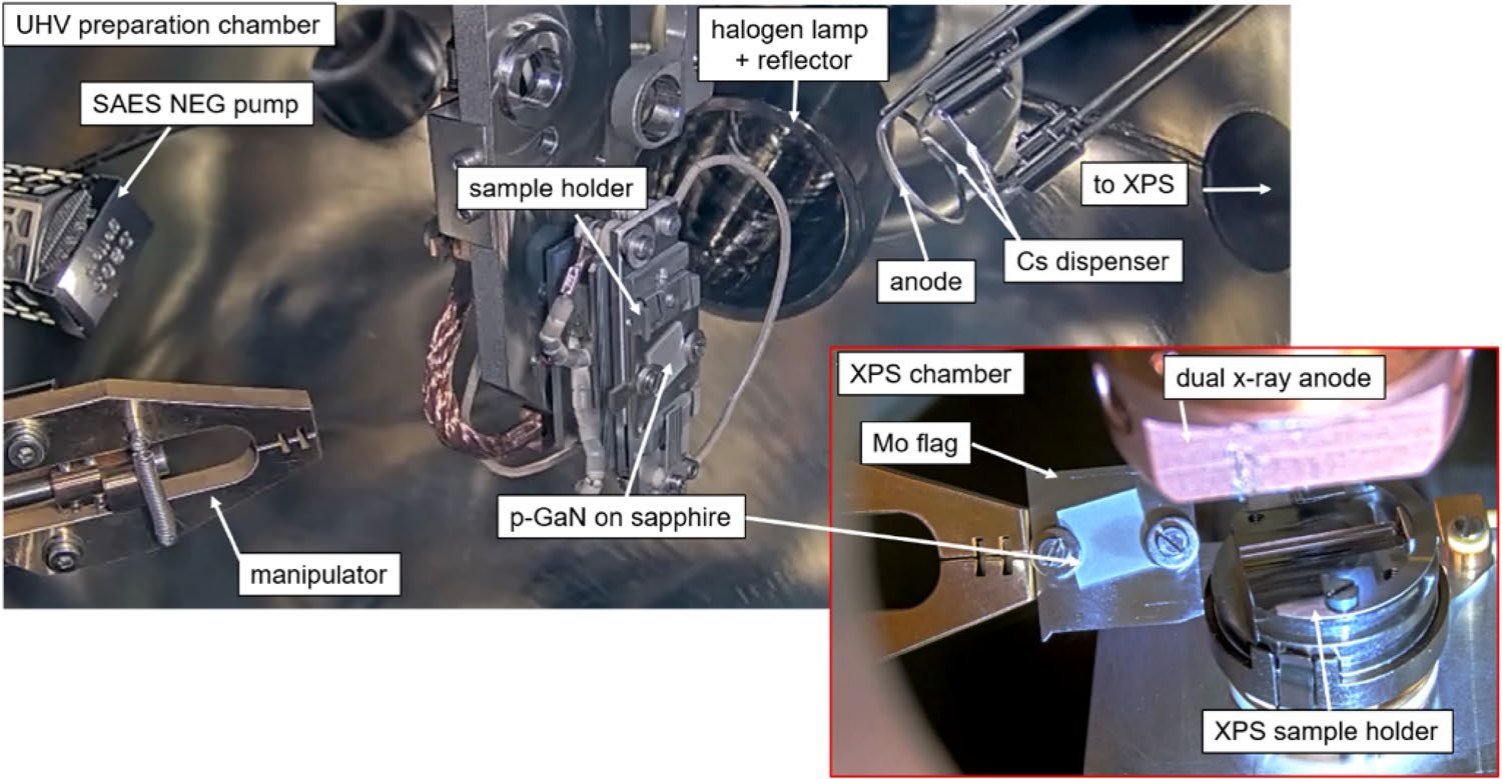
The interior of the UHV preparation chamber (showing a sample holder, a halogen lamp with reflector, a steel ring anode, and Cs dispensers) and the XPS analysis chamber connected to the preparation chamber.

Each EtOH-cleaned p-GaN sample was transferred into a glovebox in a dry nitrogen atmosphere. It was mounted on the sample holder (molybdenum flag) and transported in a dry nitrogen atmosphere into a UHV chamber, as shown in Fig. 11. In the UHV chamber, the sample underwent a thermal cleaning using a 400 W halogen lamp with a stainless-steel reflector. The temperature on the p-GaN surface was measured with an infrared (IR) sensor calibrated by the supplier for measurements on semiconductor surfaces.

For the Cs deposition dispensers from SAES were used that released a constant Cs flux at 3.3–3.5 A, causing a pressure of about 1 × 10^−9^ mbar.

The activation was conducted at room temperature solely with Cs, while the p-GaN sample was illuminated by UV light at a 310 nm wavelength and 110 µW maximum output power. An aperture in front of the UV-LED controlled the LED’s output light power. The extracted photoelectrons from the p-GaN:Cs photocathode were collected by a steel ring anode with a positive bias of 200 V. The photocurrent during the Cs deposition was recorded in-situ, and the deposition was stopped when the photocurrent plateaued at a maximum value.

The QE was calculated by Eq. (1), where *h* is the Planck constant, *c* is the speed of light, *q*_e_ is the elementary charge of an electron, *λ* is the wavelength, and *P*_Light_ is the power of the drive light on the cathode, and *I* is the photocurrent of the p-GaN:Cs photocathode^34^.

1 $$ {\text{QE}} = \frac{h \cdot c}{{q_{{\text{e}}} }} \cdot \frac{I}{{\lambda \cdot P_{{{\text{Light}}}} }} $$

### Instrumental parameters of in-situ XPS

The UHV preparation chamber was directly connected to an X-ray photoelectron spectrometer (XPS), as shown in Fig. 11, and the sample surface was studied after each treatment step at room temperature. The sample was transported under UHV conditions from the preparation chamber into the XPS chamber via a manipulator. The XPS sample holder was a home-built construction fitting the molybdenum flag with the sample on top.

XPS experiments were performed by an electron spectrometer (PHI 5600) at an average pressure of 5 × 10^–9^ mbar. The XPS spectra were conducted using a non-monochromatized dual X-ray source with an Al Kα line (*hv* = 1486 eV) and an Mg Kα line (*hv* = 1254 eV) and a 4 mm aperture. The XPS spectra were collected using a commercial system equipped with a hemispherical energy analyzer. Survey spectra were taken with a pass energy of 117.4 eV and a step energy of 1.0 eV. Detailed spectra were taken with a pass energy of 58.8 eV and an energy step of 0.25 eV. The energy scale was calibrated to the peak position of the Cu 2p_*3/2*_ (932.4 eV) and Au 4f_*7/2*_ (83.9 eV). The the full-width half-maximum (FWHM) bandwidth of Cu 2p_*3/2*_ was 1.19 eV and the FWHM of Au 4f_*7/2*_ was 1.30 eV after ion cleaning. The fitting of the experimental data was obtained using Casa XPS and a Lorentzian asymmetric (LA) line shape (1.53, 243) and a Shirley background was applied to all spectra.

## Supplementary Information

Supplementary Information.

## Data Availability

The datasets used and analysed during the current study is available from the corresponding author on reasonable request.
