# Supplementary material for: Influence of surface carbon on the performance of cesiated p-GaN photocathodes with high quantum efficiency
Source: Sci Rep. 2023 Feb 23;13:3188. doi: 10.1038/s41598-023-30329-0 (PMC9950145; doi:10.1038/s41598-023-30329-0)
Supplement: Supplementary file 1 — Supplementary Information. [file 41598_2023_30329_MOESM1_ESM.docx]

# Supplementary


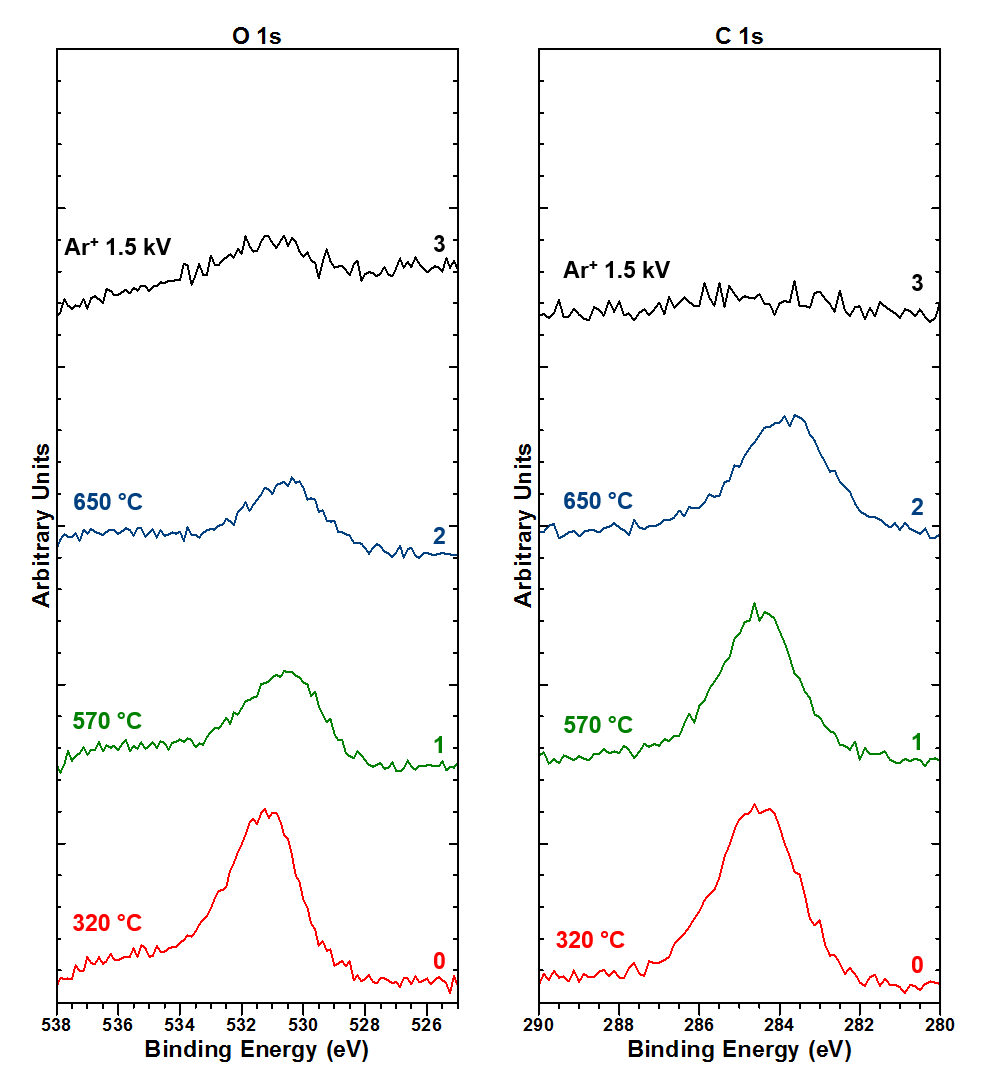


**Fig. S1.** O 1s and C 1s photoelectron spectra for the p-GaN surface thermal cleaned at 320 °C (line 0), 570 °C (line 1), 650 °C (line 2) and after Ar^+^ irradiation (line 3).

XPS studies of the activation and degradation was repeated for two other p-GaN on sapphire samples (sample B and C) to confirm the observed effects on sample A and to improve the statistic accuracy.


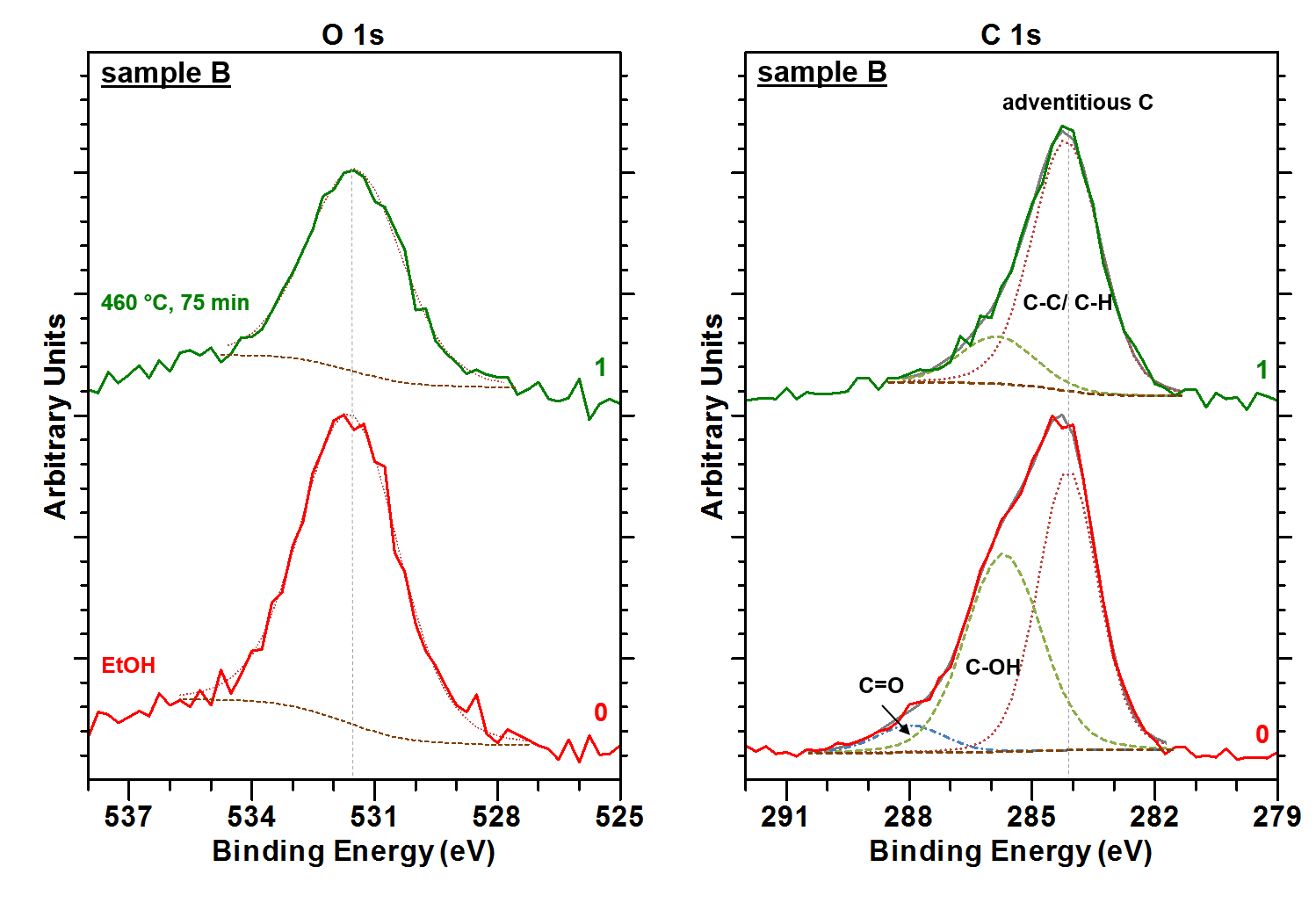


|  |
| --- |
| **Fig. S2.** O 1s and C 1s photoelectron spectra for the p-GaN surface (sample B) cleaned with EtOH (line 0) and after thermal cleaning at 460 °C (line 1). |

| 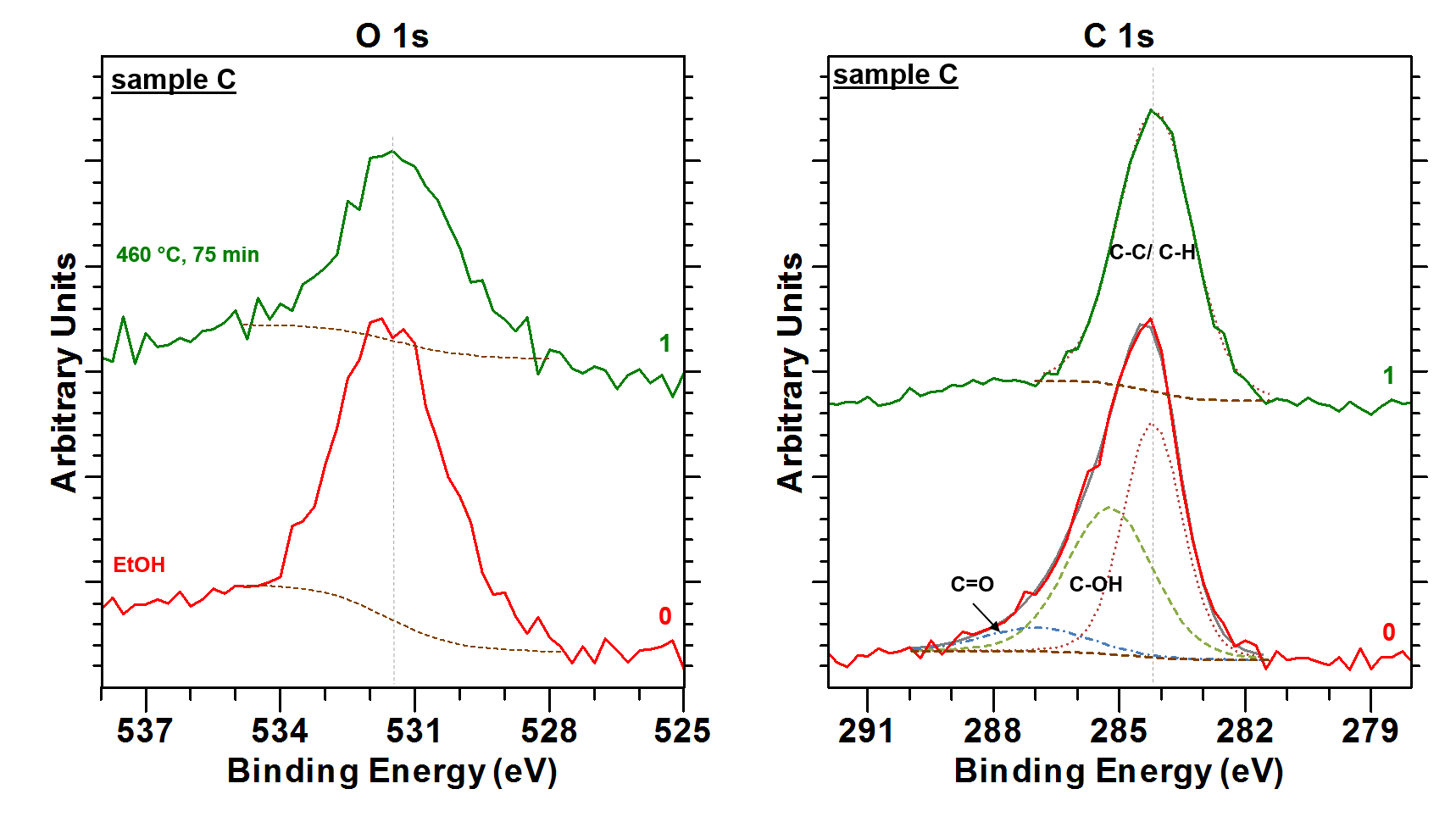 |
| --- |
| **Fig. S3.** O 1s and C 1s photoelectron spectra for the p-GaN surface (sample C) cleaned with EtOH (line 0) and after thermal cleaning at 460 °C (line 1). |

| 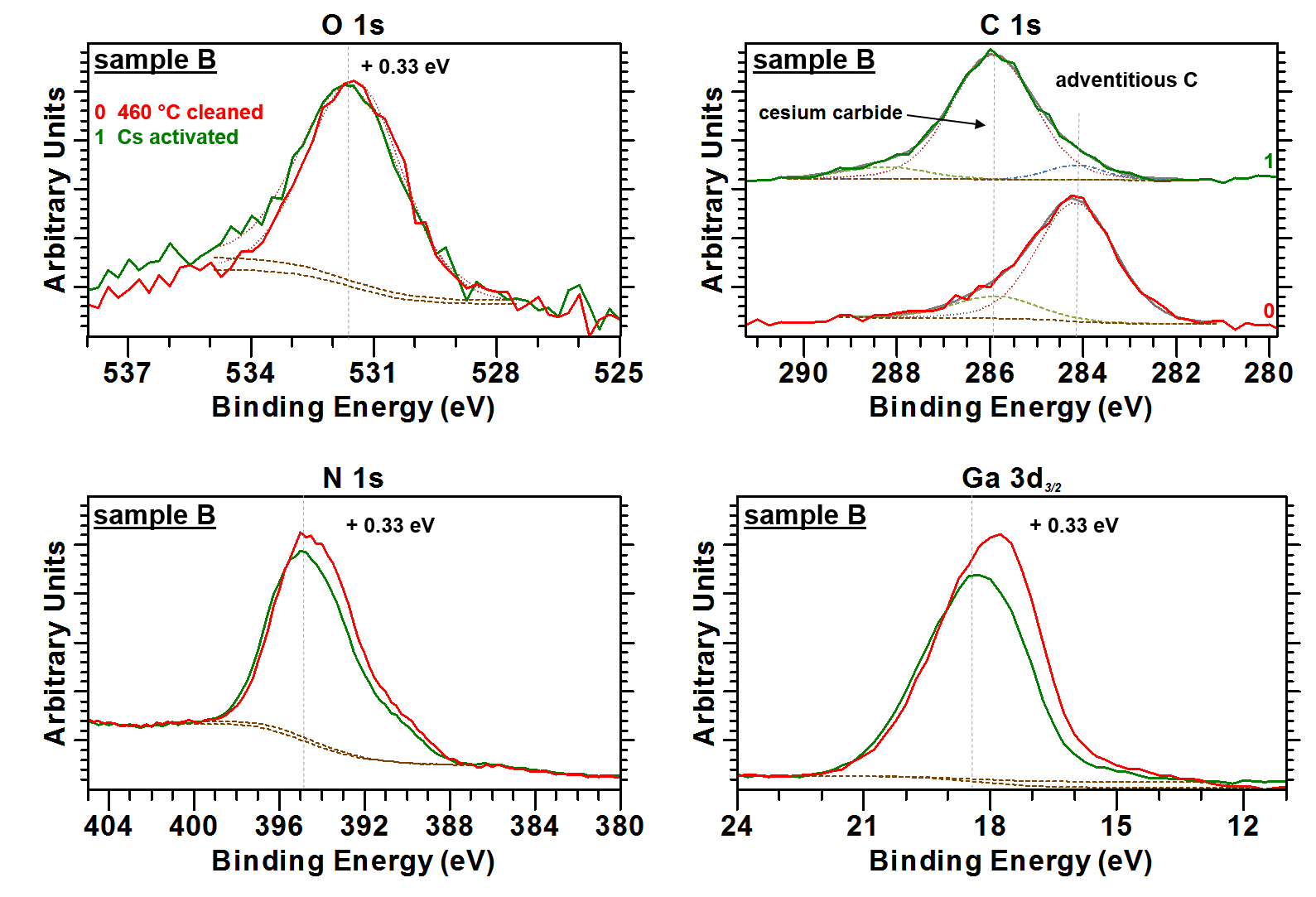 |
| --- |
| **Fig. S4.** Ga 3d*_3/2_*, N 1s, O 1s and C 1s photoelectron spectra for the p-GaN surface (sample B) after thermal cleaning at  460 °C (line 0) and after Cs activation with 3.7 % QE (line 1). |

| 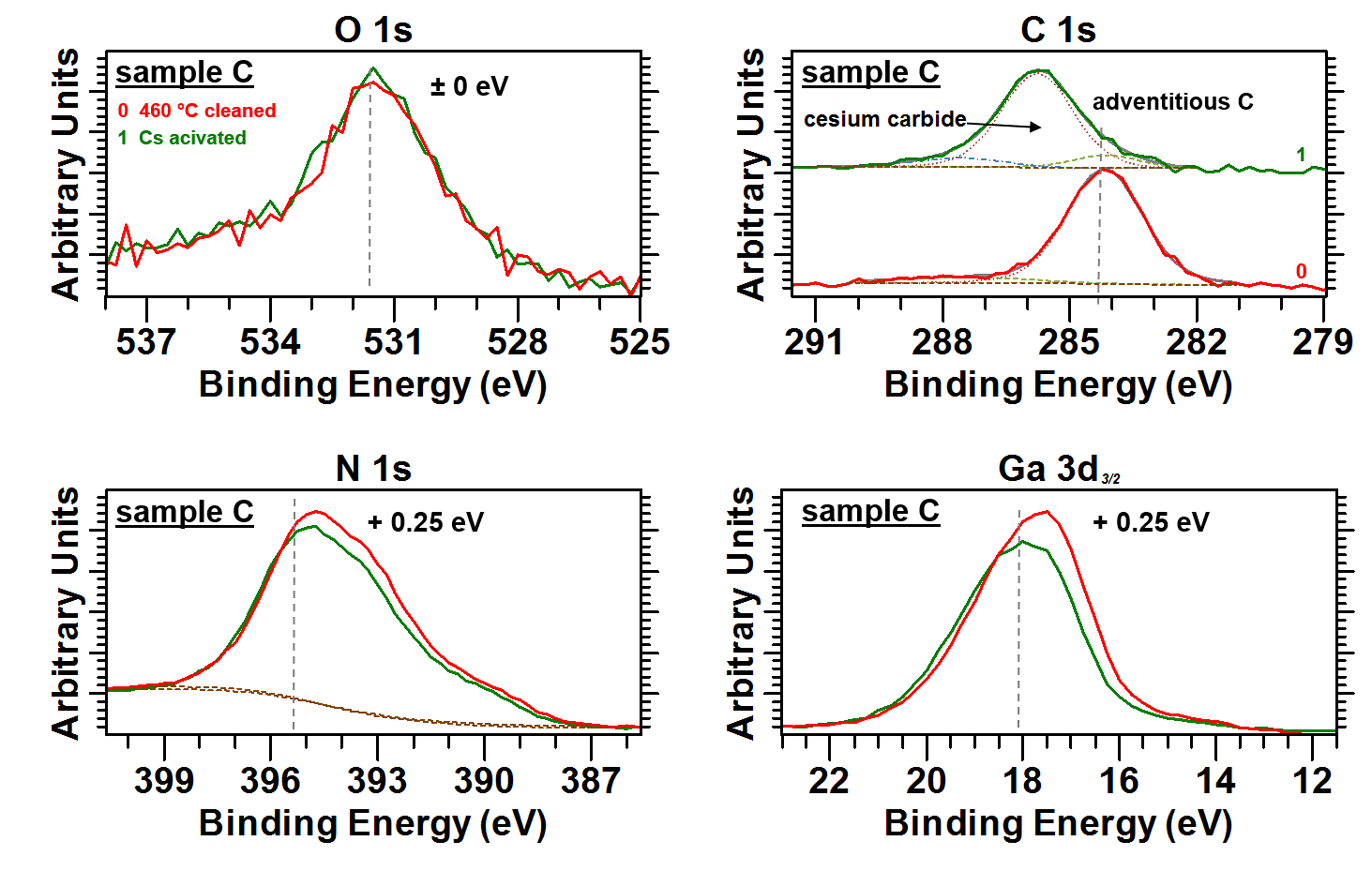 |
| --- |
| **Fig. S5.** Ga 3d*_3/2_*, N 1s, O 1s and C 1s photoelectron spectra for the p-GaN surface (sample C) after thermal cleaning at  460 °C (line 0) and after Cs activation with 6.0 % QE (line 1). |

| 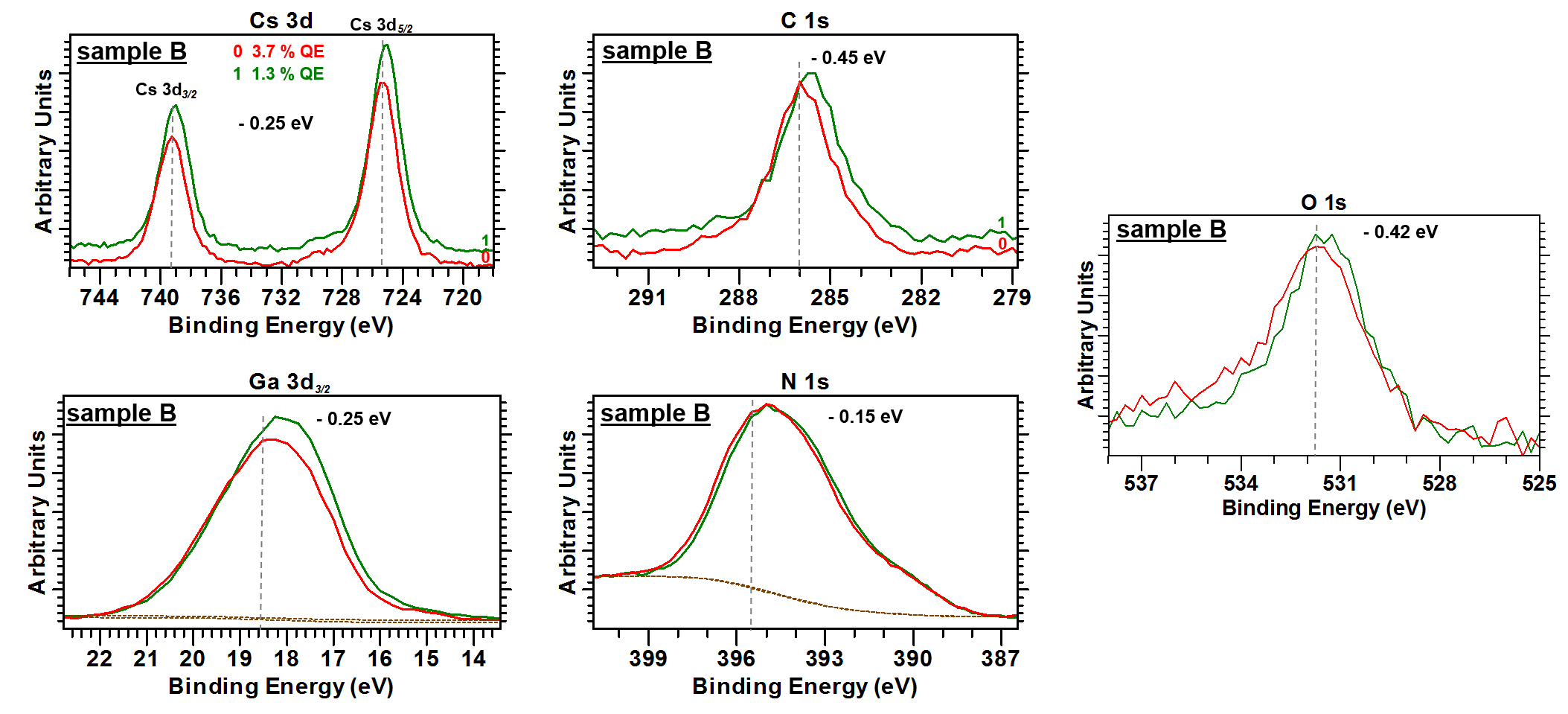 |
| --- |
| **Fig. S6.** Ga 3d*_3/2_*, Cs 3d, N 1s, O 1s and C 1s photoelectron spectra for the p-GaN surface (sample B) after Cs activation with 3.7 % QE (line 0) and with 1.3 % QE (line 1). |

| 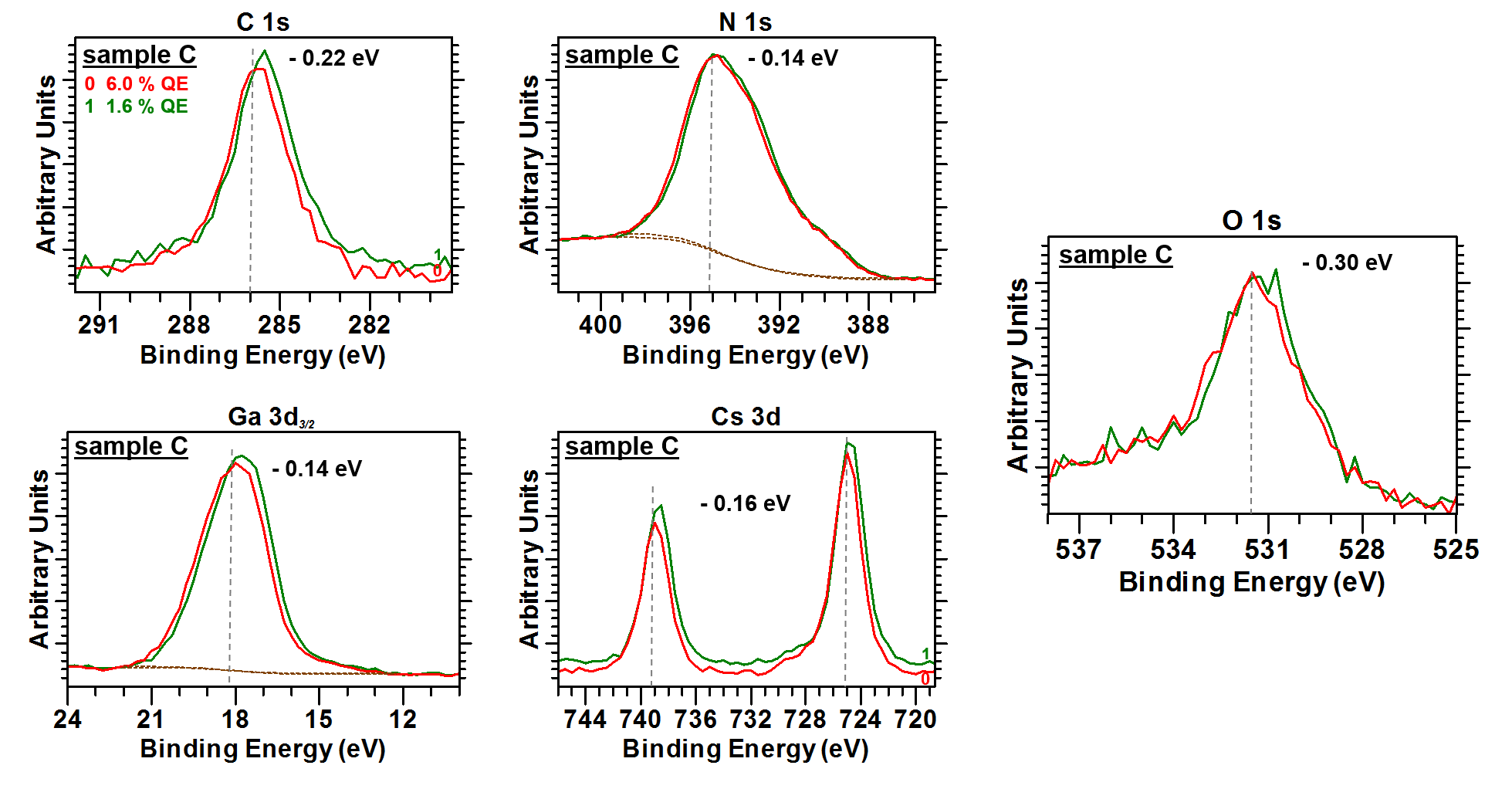 |
| --- |
| **Fig. S7.** Ga 3d*_3/2_*, Cs 3d, N 1s, O 1s and C 1s photoelectron spectra for the p-GaN surface (sample C) after Cs activation with 6.0 % QE (line 0) and with 1.6 % QE (line 1). |
